# The Changes of Phyllosphere Fungal Communities among Three Different *Populus* spp.

**DOI:** 10.3390/microorganisms11102479

**Published:** 2023-10-02

**Authors:** Zhuo Sun, Weixi Zhang, Yuting Liu, Changjun Ding, Wenxu Zhu

**Affiliations:** 1 College of Forestry, Shenyang Agriculture University, Shenyang 110000, China; sunzhuo13213924090@163.com (Z.S.); 2021240803@test.syau.edu.cn (Y.L.); 2 State Key Laboratory of Tree Genetics and Breeding, Research Institute of Forestry, Chinese Academy of Forestry, Beijing 100083, China; weixizhang@caf.ac.cn; 3 Key Laboratory of Tree Breeding and Cultivation of State Forestry Administration, Research Institute of Forestry, Chinese Academy of Forestry, Beijing 100083, China; 4 Research Station of Liaohe-River Plain Forest Ecosystem, Chinese Forest Ecosystem Research Network (CFERN), Shenyang Agricultural University, Tieling 110161, China

**Keywords:** fungal community, high-throughput sequencing, leaf characteristics, microorganism, phyllosphere, *Populus* spp.

## Abstract

As an ecological index for plants, the diversity and structure of phyllosphere microbial communities play a crucial role in maintaining ecosystem stability and balance; they can affect plant biogeography and ecosystem function by influencing host fitness and function. The phyllosphere microbial communities reflect the immigration, survival, and growth of microbial colonists, which are influenced by various environmental factors and leaves’ physical and chemical properties. This study investigated the structure and diversity of phyllosphere fungal communities in three different *Populus* spp., namely—*P*. *× euramaricana* (BF3), *P. nigra* (N46), and *P. alba × P. glandulosa* (84K). Leaves’ chemical properties were also analyzed to identify the dominant factors affecting the phyllosphere fungal communities. N46 exhibited the highest contents of total nitrogen (Nt), total phosphorus (Pt), soluble sugar, and starch. Additionally, there were significant variations in the abundance, diversity, and composition of phyllosphere fungal communities among the three species: N46 had the highest Chao1 index and observed_species, while 84K had the highest Pielou_e index and Simpson index. Ascomycota and Basidiomycota are the dominant fungal communities at the phylum level. Results from typical correlation analyses indicate that the chemical properties of leaves, especially total phosphorus (Pt), total nitrogen (Nt), and starch content, significantly impact the structure and diversity of the phyllosphere microbial community. However, it is worth noting that even under the same stand conditions, plants from different species have distinct leaf characteristics, proving that the identity of the host species is the critical factor affecting the structure of the phyllosphere fungal community.

## 1. Introduction

The phyllosphere is the interface between the aerial part of a plant and the air, housing many microorganisms such as bacteria, fungi, and archaea collectively called phyllosphere microorganisms [1]. These microorganisms are critical in the carbon and nitrogen cycle. Their relationship with the host plant is complex, and mostly mutually beneficial [2]. These microorganisms can affect the growth and ecological function of the host plant in many ways. Ursula et al. [3] discovered that phyllosphere bacteria can improve strawberry transpiration. It has been suggested that some genera of *Staphylococcus*, *Sphingomonas*, and *Polaromonas* can degrade organic pollutants in the atmosphere [4,5,6]. Moreover, some fungi modulate their physiological and metabolic functions to support plant stress tolerance [7]. For instance, Bargabus RL et al. [8] reported that *Bacillus mycoides* can improve the activity of sugar beet self-related defense enzymes (chitinase and peroxidase) and induce the plant to activate systemic resistance to defend against beet tail leaf spot. 

Environmental factors, such as precipitation, wind, and solar radiation, have been shown to play an essential role in determining patterns of phyllosphere microbial community colonization [9]. Microbial communities undergo natural selection in every ecosystem, leading to variations in microbial composition under different circumstances [10,11], suggesting that environmental forces play a significant part in introducing microorganisms to the plant phyllosphere. At the same time, phyllosphere microbial communities have the potential to influence plant biogeography and ecosystem function through their influence on plant performance under different environmental conditions [12,13,14]. However, several authors demonstrated that regardless of the geographic location, the phyllosphere microbes of the same pine species were highly similar [15,16,17]. Therefore, the factors determining the composition of plant phyllosphere microbial community composition are not environmental, but plant-specific [18,19,20]. Different host plant species can create diverse microenvironments, including different host organs, plant external tissues (epiphytes), and internal tissues (endophytes), which can affect the growth and development of various microorganisms that inhabit them [21,22,23]. 

Phyllosphere microbial communities vary among and within species [24]. However, the differences between the different plant species are mainly related to the differences in the physical and chemical properties of plant leaves. Plants have varying leaf characteristics such as stomatal density, water content, nutrient composition (carbon, phosphorus, nitrogen, starch, and soluble sugar), and leaf thickness [25,26,27]. Yadav et al. found that differences in resident populations between different plant species can be attributed to various plant physiochemical characteristics, such as the water and phosphorus contents of leaves, levels of phenolic compounds (which may inhibit many bacteria), and leaf and mesophyll thickness. Hunter PJ et al. also found that plant morphology and soluble carbohydrate content significantly affect the diversity and composition of the phyllosphere microbial community [28]. All these factors impact the colonization of phyllosphere microbial communities. However, further investigation is needed to determine the specific effects of leaf-related physicochemical properties among different *Populus* spp. in the same habitat on phyllosphere microbial communities. Therefore, conducting studies that compare the differential phyllosphere fungal communities of various *Populus* spp. could provide valuable insights into how plant host-associated leaf traits shape their unique phyllosphere fungal communities and how leaf characteristics influence these communities. 

In the current study, we selected three different *Populus* spp. in the same habitat, namely—*P*. *× euramaricana* (BF3), *P. nigra* (N46), and *P. alba × P. glandulosa* (84k), from Beixindian village Seed Science and Technology Park as the experimental subjects and used MiSeq high throughput sequencing to better understand the differences of fungal communities of different *Populus* spp. Our results provide novel evidence of the drivers of phyllosphere fungal community assemblage among different *Populus* spp. by revealing the differences in the structure and diversity of phyllosphere fungal communities of different poplar varieties under the same geographical environment.

## 2. Materials and Methods

### 2.1. The Experimental Field Site 

The experimental field site was the International Seed Technology Park located in Beixindian Village in Beijing (116°40′46″ E, 39°42′59″ N). The species tested were *P*. × *euramaricana* (BF3), *P. nigra* (N46), and *P. alba* × *P. glandulosa* (84k). The terrain at the experimental site is flat, with each species occupying a 30 × 30 m plot. The experimental field site is classified as a temperate continental monsoon climate influenced by winter and summer winds. The weather conditions vary throughout the year, with the spring season characterized by arid and windy conditions while higher temperatures and increased precipitation accompany the summer. In autumn, the climatic conditions are typically crisp, while winter is cold. The annual mean temperature at the location reaches 11.3 °C, and the average precipitation is approximately 620 mm.

### 2.2. Collection and Processing of the Samples

Three 10 *×* 10 m plots were established in each plot in September 2022. Trees with similar growth patterns were selected using the five-point sampling method in each plot. Plant samples were collected from the middle of the canopy in three directions (120°) and mixed with a replicate in each sample plot. Nine leaf samples (three replicates *×* three tree species) were collected. Every leaf sample was placed in a sterile sampling bag and immediately stored at 4 °C. Phyllosphere fungal communities were analyzed according to Ren et al. [29]. Briefly 10 g of leaves from each replicate were cut into pieces and submerged in a phosphate-buffered solution (pH 7.4, 20 mL, PBS, 0.01 M). Leaves were then subjected to sonication (6 min, 40 kHz ultrasonic bath), oscillation (20 min at 200 r/min), and sonication (3 min, 40 kHz). Fungi from the oscillating liquid were collected on a 0.22 µm filter membrane and placed into sterile centrifuge tubes. The membrane samples were stored –80 °C. 

### 2.3. DNA Extraction and Fungal ITS rRNA Gene Amplification

DNA was extracted using the Fast ^®^DNA SPIN kit (MP Biomedicals, Irvine, CA, USA) following the manufacturer’s instructions. The quantity and purity of DNA were assessed using a NanoDrop NC2000 spectrophotometer (Thermo Fisher Scientific, Waltham, MA, USA). PCR amplification of the fungal ITS rRNA genes V1 region was carried out using forward primer ITS5 (F:GGAAGTAAAAGTCGTAACAAGG) and reverse primer ITS2 (R:GCTGCGTTCTTCATCGATGC), as described by Wiesmann Crispin et al. [30]. The PCR reactions used a 25 μL mixture, including Q5 reaction buffer (5×), 5 μL Q5 High-Fidelity GC buffer (5×), 2 μL dNTPs (2.5 mM), 1 mL forward primer (10 μM), 1 mL reverse primer (10 mm), 2 mL DNA template, 8.75 mL ddH_2_O, and 0.25 mL Q5 DNA polysaccharase. The amplification parameters used were as follows: initial denaturation at 98 °C for 2 min, followed by 25–30 cycles of denaturation at 98 °C for 15 s, annealing at 55 °C for 30 s, extension at 72 °C for 30 s, and a final extension step at 72 °C for 5 min and then held at 10 °C. Agencourt AMPure Beads (Beckman Coulter, Brea, CA, USA) was used to purify the PCR amplicons, and PicoGreen dsDNA Assay Kit (Invitrogen, Carlsbad, CA, USA) was used to quantify the PCR amplicons further. PCR amplicons were sequenced at Shanghai Personal Biotechnology Co., Ltd. (Shanghai, China) using the Illlumina NovaSeq platform. The high-throughput sequencing raw data of phyllosphere fungi were uploaded to NCBI database with the accession number PRJNA924105.

### 2.4. Determination of Leaves Chemical Properties

Plant leaves were repeatedly rinsed in sterile distilled water and surface-dried in phosphate-free filter paper. Samples were dried (65 °C, over 48 h, constant weight) and then baked (105 °C, 30 min). The desiccated plant foliage samples were pulverized and sifted through a 100-mesh screen. Subsequently, selected blade samples weighing approximately 3.5–4.2 mg were allocated into a tin cup (3 mm *×* 5 mm), wrapped and positioned within an autosampler. Carbon and nitrogen contents were determined using an elemental analyzer (Elementar Vario EL III Germany). Phosphorus content was analyzed via the molybdenum antimony anti-colorimetric method [31]. Soluble sugars and starch contents were determined by the anthrone colorimetric method [32].

### 2.5. Statistical Analysis

ASV-level alpha diversity indices such as the Chao1 richness estimator, observed species, Simpson index, and Pielou’s evenness were computed using the ASV table in QIIME2 and represented as box plots [33,34,35]. To visualize the variations in alpha diversity among various specimen groups, the information in the table was plotted as box plots utilizing QIIME2 (2019.4) and the ggplot2 package for the R package (v3.2.0) [36]. The Kruskal-Wallis rank sum and Dunn’s test were used to confirm the significance of differences between the various groups. A Venn diagram was generated using the R package “VennDiagram” to display shared and unique ASVs among samples or groups based on the occurrence of ASVs across samples/groups, irrespective of their relative abundance [37]. The microbiota structure among different groups was evaluated for significance of differentiation using PERMANOVA (permutational multivariate analysis of variance) [38]. The scikit-bio package in Python was used to conduct the analysis of variance between groups, with 999 permutation tests. Cluster analysis was performed using the uclust function of the R package (v3.2.0) stat package, with the UPGMA algorithm as default for the Bray-Curtis distance matrix (i.e., average clustering method), and visualized through the ggtree package script in the R package (v3.2.0) [39]. The QIIME2 (2019.4) command “qiime taxa barplot” was used to generate visualizations of the compositional distribution of each sample at four taxonomic levels (phylum, order, family, and genus). This was achieved by counting the number of features after removing singletons. The linkages between leaf chemical properties and phyllosphere fungal community composition and diversity were performed by redundancy analysis (RDA) via Canoco 5 [40]. One-way ANOVA was utilized to analyze significant differences in leaf chemical properties, and post-tests were conducted using the S-N-K q test. Significant differences in leaf chemical properties were processed in Excel (2019) and analyzed using SPSS 26.0 (Chicago, USA). 

## 3. Results

### 3.1. Changes in the Chemical Traits of the Leaves in Three Different Populus spp.

The three analyzed species of *Populus* spp. showed significant differences in concentration of TC(F = 20.12, *p* < 0.001) Nt(F = 10.58, *p* = 0.004) C/N(F = 9.86, *p* = 0.005), and Pt(F = 94.07, *p* < 0.001). The sample from 84K had the highest TC concentration (467.34 g·kg^−1^), followed by N46 (443.8 g·kg^−1^), and BF3 (439.26 g·kg^−1^). N46 had the highest concentration of soluble sugars and starch (24.08 mg·kg^−1^ and 74.13 mg·kg^−1^, respectively). The highest C/N was registered in 84K (17.79) (Table 1).

### 3.2. Diversity of Phyllosphere Fungal Communities in Three Different Populus spp.

**Figure 1:**
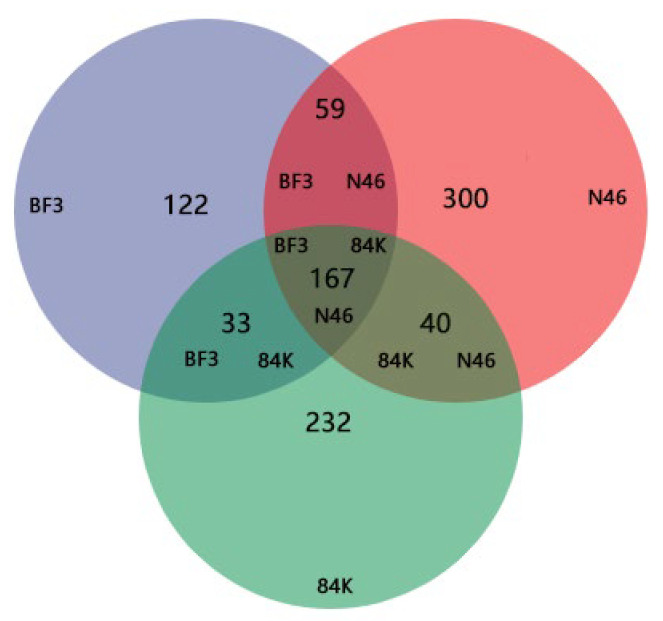
The shared and unique ASVs of phyllosphere fungi. (BF3, *P.* × *euramaricana* N46, *P. nigra* 84K, *P. alba* × *P. glandulosa*).

**Figure 2:**
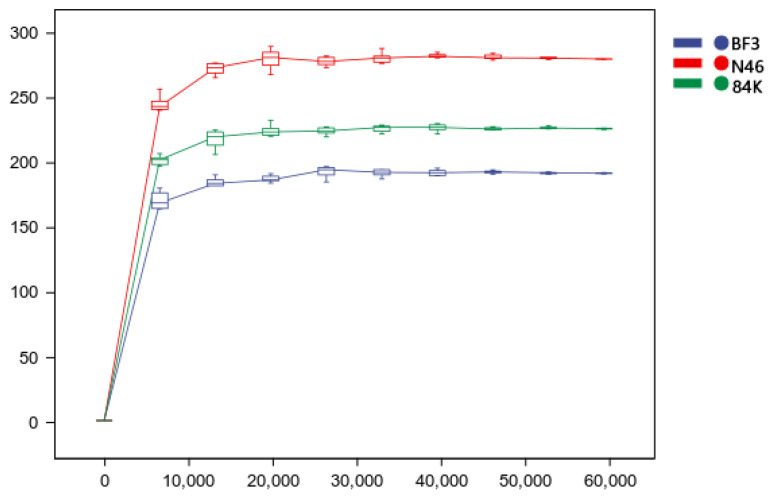
Rarefaction Curve. The flattening of the curve reflects the sequencing depth’s influence on the observed samples’ diversity. The flatter curve indicates that the sequencing results are sufficient to reflect the diversity in the current samples and many new ASVs; otherwise, it indicates that the alpha diversity is not approaching saturation.

A total of 831,838 high-quality fungal sequences were obtained. The average number of fungal sequences per sample was 69,320, comprising a clustering of 953 ASVs. The ASVs identified were 381 for BF3, 566 for N46, and 472 for 84K. The number of shared ASVs between all species was 167. The number of unique ASVs was 122 for BF3, 300 for N46, and 232 for 84K. Among the analyzed species, 1,213 shared phyllosphere fungi ASVs were identified (Figure 1). We observed a plateau in the curve with the sequencing depth increased over time (Figure 2). 

**Figure 3:**
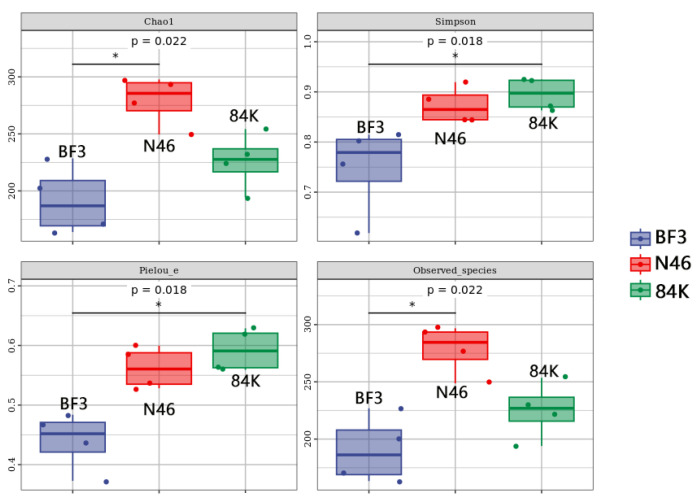
Phyllosphere fungal community diversity. (BF3, *P.* × *euramaricana* N46, *P. nigra* 84K, *P. alba* × *P. glandulosa*) * denotes significant difference at 0.05 level.

**Figure 4:**
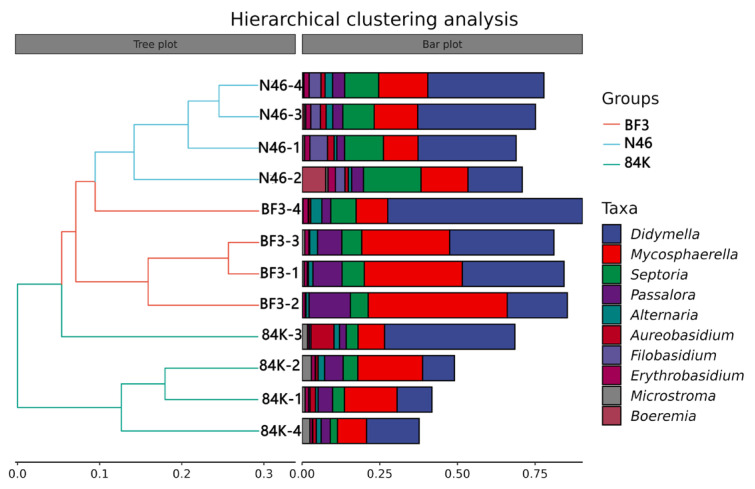
Hierarchical clustering analysis of phyllosphere fungal communities of different *Populus* spp. at the genus level (BF3, *P.* × *euramaricana* N46, *P. nigra* 84K, *P. alba* × *P. glandulosa*).

The alpha diversity indices of three different species of *Populus.* presented significant variations in phyllosphere fungal Simpson (*p* = 0.022), observed species (*p* = 0.022), Chao 1 (*p* = 0.018) and Pielou_e (*p* = 0.018) among BF3, N46, and 84K. The Simpson index and Pielou_e index were the highest in 84K (0.90 and 0.59, respectively). However, the Chao 1 index and observed species index were highest in N46 (279.59 and 278.62, respectively). The Chao1 index, Simpson index, observed species index, and Pielou_e index were the lowest in BF3 (191.58, 0.75, 190.68, and 0.44, respectively) (Figure 3). Hierarchical clustering analysis of phyllosphere fungal communities revealed that BF3 and N46 were clustered into one category (Figure 4).

### 3.3. Composition of Phyllosphere Fungal Communities in Three Different Populus spp.

**Figure 5:**
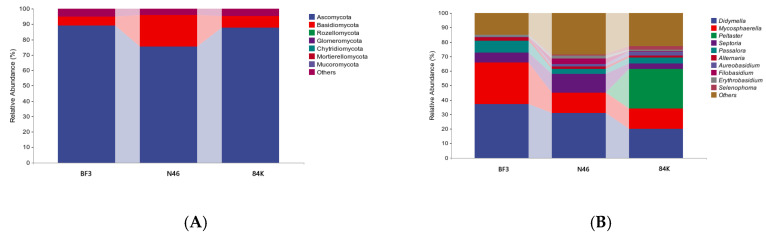
The relative abundance of phyllosphere fungal community composition at phylum (**A**) and genus level (**B**). (BF3, *P*. *× euramaricana* N46, *P. nigra* 84K, *P. alba × P. glandulosa*).

Across all samples, 36 fungal phyla were identified, including Ascomycota, Basidiomycota, Rozellomycota, and Glomeromycota, with relative abundance greater than 1% (Figure 5). Ascomycota was the dominant fungal community (84.11%), followed by Basidiomycota (11.26%). The relative abundance of Ascomycota was highest in BF3 (89.15%), followed by 84K (87.77%) and N46 (75.40%). The relative abundance of Basidiomycota was highest in N46 (20.61%). 

**Figure 6:**
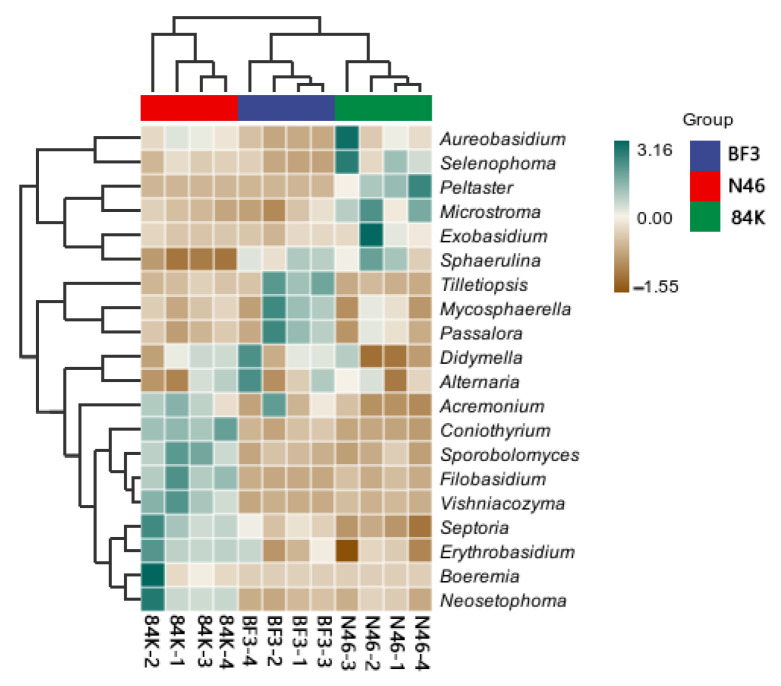
Heatmap of phyllosphere fungal communities at the top 50 at the genus level. The scale “−3 to +3” refers to the relative abundance of the corresponding taxonomic unit in each sample/group of the grouping scheme.

We identified 888 fungal genera at the genus level, including Didymella (29.42%), Mycosphaerella (18.88%), Peltaster (9.91%), Septoria (7.84%), Passalora (5.21%), Alternaria (1.78%), and Aureobasidium(1.56%) (Figure 5A). The relative abundance of Mycosphaerella and Didymella was the highest in BF3 and N46, while Peltaster was the dominant group in 84K (Figure 5B). The heatmap analysis revealed significant differences in phyllosphere fungal communities among the analyzed *Populus* spp. (Figure 6).

### 3.4. The Relationships between the Chemical Traits of the Leaves and Phyllosphere Fungal Community Composition

**Figure 7:**
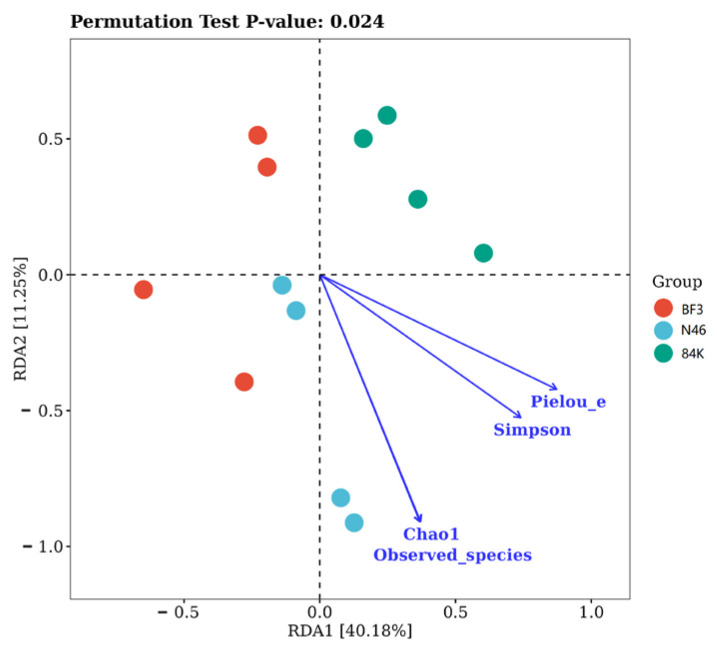
Redundancy analysis (RDA) of leaf nutrient factors and alpha diversity of phyllosphere fungal communities.

The RDA (Figure 7) revealed that phyllosphere fungal Chao 1 (r = 0.599, *p* < 0.05), observed_species (r = 0.601, *p* < 0.05), Simpson (r = 0.696, *p* < 0.05), and Pielou_e (r = 0.714, *p* < 0.01) increased with increase in starch. The Chao1 index (r = 0.736, *p* < 0.01) and the observed_species(r = 0.736, *p* < 0.01) increased with the increase in Pt content, which showed a significant positive correlation with Pt (Table 2). The relative abundance of Ascomycota decreased with the increase in Pt and starch (r = −0.830, *p* < 0.01; r = −0.738, *p* < 0.01), but increased with the increase in C/N. The relative abundance of Basidiomycota increased with the increase of Nt, Pt, and starch (r = 0.752, *p* < 0.01) (r = 0.929, *p* < 0.01; r = 0.650, *p* < 0.05) and decreased with the increase in C/N content(r = −0.717, *p* < 0.01) (Table 3).

## 4. Discussion

Previous studies have shown that after microbial cells or propagules arrive on the leaf surface, various factors determine whether they can colonize the leaf and where they become located. The establishment process is determined by interactions between leaf characteristics and environmental conditions that influence the phyllosphere habitat. Plant species have different physiological structures and leaf environments for phyllosphere microorganisms [2,41]. The species of the host plants play a critical role in determining the colonization and establishment of phyllosphere microbial communities [15,16,17]. In the present study, the three *Populus* spp.used namely—*P*. × *euramaricana* (BF3), *P. nigra* (N46), and *P. alba* × *P. glandulosa* (84k), come from the same geographical conditions. However, we found differences in the composition and structure of phyllosphere fungal communities, which may prove that host plant species have greater genetic control of phyllosphere microorganism behavior. Many of the changes in the composition of phyllosphere microbial communities can be explained by the properties of hosts [42].

Differences in the diversity of phyllosphere microbial communities among different plant species can be attributed to variations in leaf characteristics [25], nutrient concentrations or secondary plant metabolites [43]. Secondary metabolites include alkaloids, isoprenoids, and phenolic acids (leached or excreted from the leaf interior onto the surface) [44,45,46]. Tree species have a significant impact on leaf characteristics. These leaf characteristics may be different in different tree species. The phyllosphere microbial communities are associated with many leaf characteristics, including chemistry and structure. Our study found significant differences in several leaf chemical characteristics among the three *Populus* spp. including TC (F = 20.12, *p* < 0.001), Nt (F = 10.58, *p* = 0.004), Pt (F = 94.07 *p* < 0.001), C/N (F = 9.86, *p* = 0.005), soluble sugar (F = 1.04, *p* = 0.392), and starch (F = 2.45, *p* = 0.142), among BF3, N46, and 84K. Specifically, Nt, Pt, soluble sugar, and starch contents were highest in N46. In contrast, 84K had the highest TC and C/N contents (Table 1). These results indicate that tree species significantly impact leaf characteristics, leading to changes in phyllosphere fungal communities of different host plant species. 

Current studies have shown that nutrient concentrations on the leaf surface influence the characteristics of the phyllosphere fungal communities. The release of nutrients can support microbial growth. The nutrients on the surface of leaves, such as sugar and minerals may vary depending on the tree species [47]. A correlation analysis between physicochemical properties and alpha diversity of the phyllosphere fungal community revealed that leaf phosphorus, nitrogen, and starch contents were the main influences on the alpha diversity index (Table 2). This is similar to the findings of Kembel et al., where leaf nitrogen and phosphorus content affected the phyllosphere fungal communities [27]. This correlation may be explained by the structural characteristics of the leaves, such as the cuticle covering the epidermal cell walls of higher plants, which remarkably affect the phyllosphere microbial colonization. Cuticular waxes can limit nutrient diffusion and reduce leaf-surface wetting [48,49,50]. The cuticle-mediated limitation of nutrient loss from the leaf is significant in supporting epiphytic microbial populations. Due to the hydrophobic nature of cutin and waxes, which are the lipid components of the cuticle, it form an effective transport barrier for water and polar substances. Only lipophilic molecules are expected to penetrate cuticles in significant amounts, which means that the phosphorus availability may limit the growth of epiphytic microbes. Our experimental results support this idea, as the content of TP was found to be significantly associated with the diversity of phyllosphere fungal communities (Table 2 and Table 3). However, relevant studies suggest that the content of soluble sugars may not significantly effect on the colonization of phyllosphere microbial communities. This may be because monosaccharides do not diffuse across intact cuticles at rates required to sustain the growth of epiphytic microorganisms. Similar results were obtained in our experiments, with no significant difference in the content of soluble sugars in BF3, N46 and 84K leaves. Leaf physicochemical properties and correlation analysis also revealed no significant correlation between soluble sugars and fungal community composition and diversity. Therefore, we can infer that the content of soluble sugars in BF3, N46, and 84K is not the main factor leading to differences in the structure and diversity of phyllosphere fungal communities among the three samples. Instead, the content of TP may be the main factor driving these differences.

High-throughput sequence analysis of ITS rDNA revealed that the dominant taxa of phyllosphere fungi are mainly Ascomycota and Basidiomycota. These findings are consistent with other scholars showing that, at the phylum level, Ascomycota and Basidiomycota are the dominant groups of phyllosphere microorganisms [27], indicating that the host plant’s selection process reduces the diversity of the phyllosphere microbial community [51]. In other words, the phylogeny of the host plant determines the degree to which the tree species are similar [52].

## 5. Conclusions

Above all, the identity of the host species significantly influences leaf characteristics. Our study found that different *Populus* spp. under the same stand conditions have different leaf characteristics, which resulted in different phyllosphere fungal communities. The abundance, composition, and diversity of phyllosphere fungal communities in BF3, N46, and 84K varied greatly in this study. Plant leaf characteristics are the key factors affecting the structure and diversity of microbial communities. This study revealed the correlation between leaf chemical characteristics and phyllosphere fungal communities. Our results can provide a reference for studying the leading factors of the differences in microbial community structure and diversity among plant species.

## Figures and Tables

**Table 1:** Variation in the chemical properties of the leaves of different species of *Populus* spp.

	Total Carbon/g kg^−1^	Total Nitrogen/g kg^−1^	C/N	Total Phosphorus/g kg^−1^	Soluble Sugar/mg kg^−1^	Starch/mg kg^−1^
BF3	439.26 ± 5.47 b	25.19 ± 0.97 b	17.51 ± 0.64 a	2.09 ± 0.16 b	15.72 ± 3.76 a	57.52 ± 4.61 a
N46	443.82 ± 0.94 b	31.37 ± 0.56 a	14.16 ± 0.28 b	6.40 ± 0.41 a	24.08 ± 5.53 a	74.13 ± 6.77 a
84 K	467.34 ± 1.75 a	26.46 ± 1.33 b	17.79 ± 0.87 a	2.10 ± 0.06 b	21.61 ± 2.89 a	68.44 ± 4.50 a
*p* value	<0.001	0.004	0.005	<0.001	0.392	0.142
F value	20.12	10.58	9.86	94.07	1.04	2.45

Mean values ± standard error. Different lower-case letters in the same column indicate a significant difference at *p* < 0.05. (BF3, *P. × euramaricana* N46, *P. nigra* 84K, *P. alba × P. glandulosa)*.

**Table 2:** The relationships between phyllosphere fungal community diversity with leaf characteristics.

Fungi	TC	Nt	C/N	Pt	Souble Sugar	Starch
Chao1	0.903	0.035 *	0.065	0.006 **	0.416	0.039 *
Observed_species	0.898	0.035 *	0.065	0.006 **	0.415	0.039 *
Pielou_e index	0.054	0.228	0.531	0.411	0.553	0.009 **
Simpson	0.143	0.165	0.351	0.453	0.903	0.012 *

TC, total carbon Nt, total nitrogen C/N, total carbon/total nitrogen ratio Pt, total phosphorus. ** denotes a significant difference at the 0.01 level; * denotes significant difference at 0.05 level.

**Table 3:** The relationships between phyllosphere fungal community composition at phylum level with leaf characteristics.

Fungi	TC	Nt	C/N	Pt	Souble Sugar	Starch
Ascomycota	0.467	0.025 *	0.034 *	0.001 **	0.289	0.006 **
Basidiomycota	0.469	0.005 **	0.009 **	0 **	0.158	0.022 *
Rozellomycota	0.114	0.326	0.497	0.882	0.540	0.830
Glomeromycota	0.903	0.227	0.253	0.068	0.768	0.342
Chytridiomycota	0.289	0.672	0.494	0.322	0.645	0.751
Mortierellomycota	0.376	0.835	0.641	0.780	0.676	0.406

TC, total carbon Nt, total nitrogen C/N, total carbon/total nitrogen ratio Pt, total phosphorus. ** denotes a significant difference at the 0.01 level; * denotes significant difference at 0.05 level.

## Data Availability

The NCBI database SRA accession number for of the raw high-throughput sequencing data is PRJNA924105.
